# Supplementary material for: Improved Long-Term Preservation of Cannabis Inflorescence by Utilizing Integrated Pre-Harvest Hexanoic Acid Treatment and Optimal Post-Harvest Storage Conditions
Source: Plants (Basel). 2024 Mar 30;13(7):992. doi: 10.3390/plants13070992 (PMC11013627; doi:10.3390/plants13070992)
Supplement: Supplementary file 1 [file plants-13-00992-s001.zip › plants-2927562-supplementary.pdf]

Table S1. Comparison between the compounds concentrations determined at harvest day (t_0) and at the end of the drying/curing phase (d6) within each of the treatment groups, namely REF (reference group) and Hex8mM (8mM hexanoic acid treatment group)[a]. Only compounds exhibiting significant differences are listed in the table.

	REF Concentration ± SE at t_0	REF Concentration ± SE at d6 after drying and curing	Hex8mM Concentration ± SE at t_0	Hex8mM Concentration ± SE at d6 after drying and curing
THC	0.17 ± 0.04	0.25 ± 0.04 (**)[a]	0.22 ± 0.03	0.28 ± 0.08 (ns)[b]
CBG	0.10 ± 0.03	0.09 ± 0.02 (ns)	0.124 ± 0.004	0.09 ± 0.01 (*)
(-)-β-pinene	0.186 ± 0.008	0.158 ± 0.003 (**)	0.18 ± 0.02	0.18 ± 0.01 (ns)
β-myrcene	0.054 ± 0.003	0.028 ± 0.001 (****)	0.050 ± 0.0027	0.035 ± 0.002 (****)
d-limonene	0.67 ± 0.02	0.428 ± 0.002 (****)	0.61 ± 0.02	0.48 ± 0.06 (****)
Linalool	0.111 ± 0.008	0.074 ± 0.003 (****)	0.10 ± 0.01	0.086 ± 0.005 (ns)
Fenchol	0.070 ± 0.004	0.057 ± 0.002 (*)	0.063 ± 0.008	0.067 ± 0.006 (ns)
Pinalol	0.076 ± 0.004	0.067 ± 0.002 (*)	0.069 ± 0.008	0.075 ± 0.005 (ns)
α-terpineol	0.077 ± 0.005	0.064 ± 0.006 (*)	0.074 ± 0.009	0.080 ± 0.006 (ns)
β-caryophyllene	0.47 ± 0.03	0.394 ± 0.006 (**)	0.39 ± 0.04	0.43 ± 0.03 (ns)
α-humulene	0.18 ± 0.02	0.149 ± 0.004 (*)	0.15 ± 0.02	0.16 ± 0.02 (ns)
(-)-α-bisabolol	0.061 ± 0.003	0.053 ± 0.002 (*)	0.053 ± 0.005	0.059 ± 0.004 (ns)
γ-elemene	0.12 ± 0.02	0.095 ± 0.001 (*)	0.089 ± 0.006	0.10 ± 0.02 (ns)
β-eudesmene	0.07 ± 0.01	0.053 ± 0.002 (*)	0.054 ± 0.006	0.057 ± 0.009 (ns)
α-selinene	0.07 ± 0.01	0.055 ± 0.002 (*)	0.056 ± 0.007	0.060 ± 0.009 (ns)

[a] Two-way ANOVA followed by Dunnet's posthoc at an adjusted significance value of $p < 0.05$: p value < 0.0332 (*), $p < 0.0021$ (**), $p < 0.0002$ (***), $p < 0.0001$ (****).
[b] ns, not significant.

Table S2. Comparison between THCA, CBGA and CBDA concentrations as a function of hexanoic acid concentration (i.e. 0 mM, 1 mM, 4 mM, 8 mM and 10 mM) at the end of the drying/curing phase (day 6) in order to determine the optimal concentration for spray application of hexanoic acid. Statistical significance for each compound was calculated using two-way ANOVA followed by Tukey's posthoc test at $\alpha = 0.05$. Letters in each row in the table are used to present the statistical test results for each compound.

	REF	Hex1mM	Hex4mM	Hex8mM	Hex10mM
THCA	15.1 ± 0.6 a	14.6 ± 0.5 a	13.2 ± 0.6 b	16.6 ± 1.3 c	14.6 ± 2.0 a
CBGA	0.64 ± 0.03 a	0.44 ± 0.02 a	0.58 ± 0.02 a	0.73 ± 0.06 a	0.30 ± 0.04 a
CBDA	0.039 ± 0.003 a	0.052 ± 0.008 a	0.054 ± 0.005 a	0.042 ± 0.003 a	0.057 ± 0.006 a

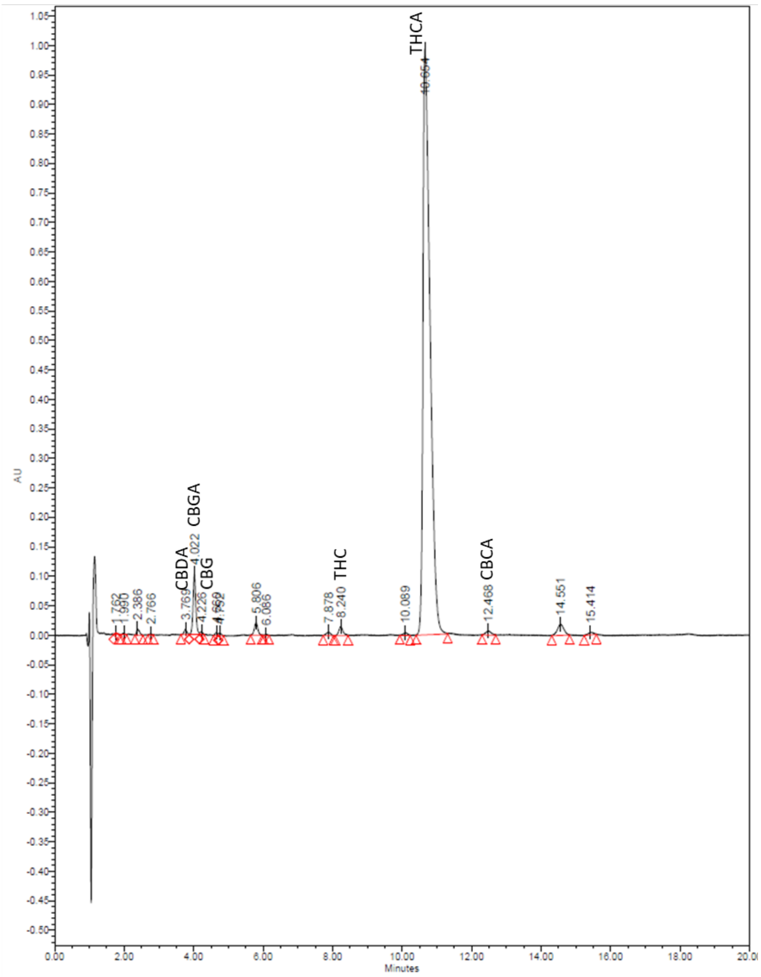

Figure S1. Representative HPLC-PDA chromatogram of the 505 chemovar ethanolic extract.

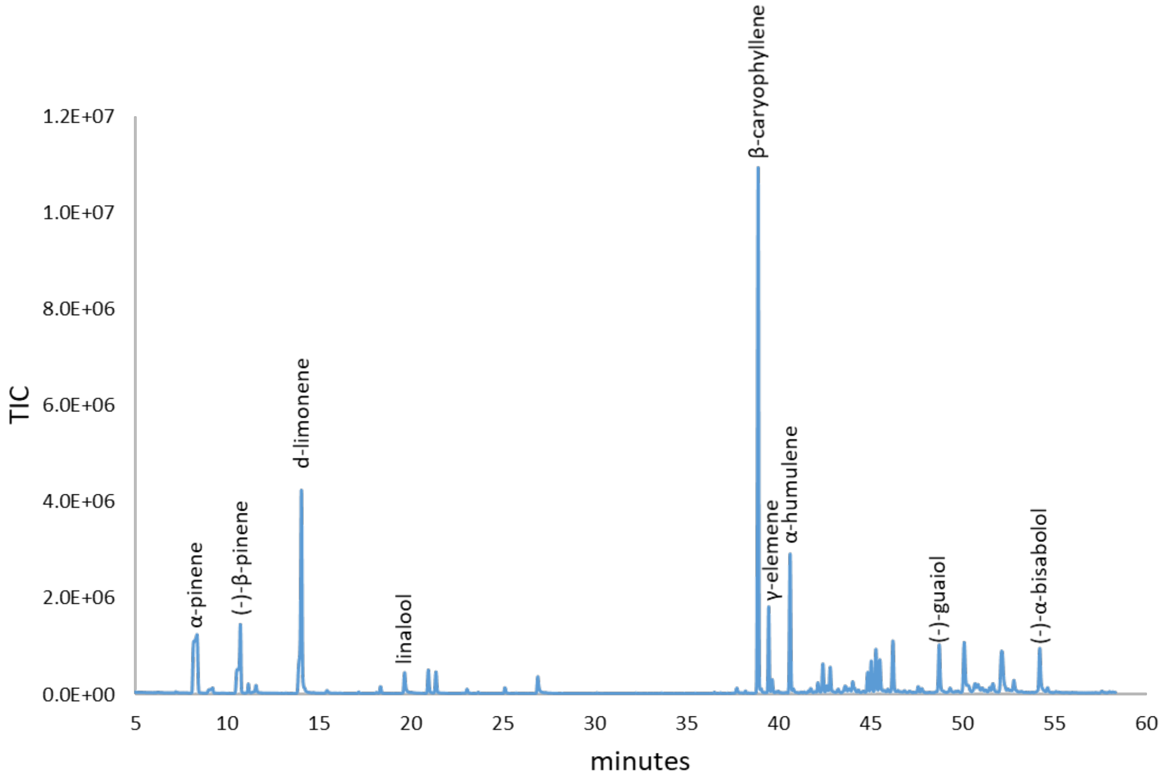

Figure S2. Representative GC/MS chromatogram of the 505 chemovar ethanolic extract.
